# Intelligent Evaluation Algorithm of English Writing Based on Semantic Analysis

**DOI:** 10.1155/2022/8955638

**Published:** 2022-10-05

**Authors:** Jing Wang, Bin Liu

**Affiliations:** ^1^School of Foreign Studies, Suqian University, Suqian 223800, Jiangsu, China; ^2^School of Education, Taylor's University, Subang Jaya 475002, Selangor, Malaysia

## Abstract

In order to solve the intelligent evaluation of English writing, this paper proposes a method based on the English semantic neural network algorithm. This paper first briefly analyzes the research background of the English semantic analysis system, then expounds on the relevant technologies of the English distance similarity algorithm, semantic analysis intelligent algorithm structure, word analysis algorithm, sentence part of speech analysis algorithm, sentence semantic analysis algorithm, and neural network algorithm, and finally expounds the database and method implementation of the English semantic analysis system, so as to provide guarantee for the design of the English semantic analysis system. The experimental results show that the recognition accuracy of the BRF network for English characters can reach 96.35%, which is 7.79% higher than that of the BP network; the AUC of the BRF network reaches 0.89, which is closer to 1 compared with 0.72 of the BP network. The test results are in good agreement with the antinoise curve test results of the figure. It is proved that the English semantic neural network algorithm can effectively improve the accuracy of English translation and further improve the efficiency of the system.

## 1. Introduction

International events and trade were expanding, and more and more attention was being paid to English as an international language. English translation has become an integral part, and all types of translators have improved rapidly [1]. The translator is not limited to the translation of a sentence or phrase, but rather the text rather than the sentence, phrase, group, or genre. From an emotional point of view, the word count can be defined as the whole text or the middle of the meaning of a word. Thus, the meaning of a word is related and has similar properties, for example, the union of two words with the same meaning [2]. The more similar the meaning of the words, the harder it is to translate. The development of an intelligent English semantic analysis algorithm based on English semantic analysis is important for the development of an English semantic analysis system model to improve the accuracy of English semantic translation [3].

Computers have been widely used to teach English. For example, many colleges, universities, and elementary and middle schools use computers to take English exams. However, the current English test only allows you to know the automatic scores of targeted questions, such as multiple-choice questions, nonwritten questions, and abbreviations punishment. Questions such as Chinese-English translation, short answers, and editing are not available. In this context, we hope that this article will improve the CAI level of English, reduce the workload of English teachers, and improve English semantic analysis based on the evaluation of intelligent writing algorithms based on English analysis [4].

## 2. Introduction to Relevant Technologies of Intelligent Evaluation Algorithm

### 2.1. English Distance Similarity Algorithm

The English language distance algorithm needs to determine the similarity of the input language. The mean value of the two terms is the mean of time [0, 1]. The correlation formula is as follows:

(1) $$\begin{array}{c} sim\left(\omega 1,\omega 2\right)=e^{-aL}\cdot \frac{1}{2}\left(\frac{Hc}{H1}+\frac{Hc}{H2}\right). \end{array}$$

In the formula, *L* is the shortest path between points *ω*1 and *ω*2; Hc is the depth of the concept word in the upper word set; depth corresponds to H1 and H2 a constant [5]. The English style, which is far from similar, can be understood as follows: the shorter the path, the shorter the two points, the deeper the similarity, and the shorter the relative length.

When measuring sentence similarity, the vector space model standard is used. The vector space model separates the smallest semantic units such as words and phrases in the text and takes the calculated similarity as vector elements. Teaching cosine is used in two English sentences to obtain semantic similarity [6, 7].

The structure of *T* is as follows:

(2) $$\begin{array}{c} T=T1\cup T2\text{,}\\ =\left\{\omega 1,\;q1,\;\ldots ,\;\omega m,\;qn\right\}\text{.} \end{array}$$

{*ω*1, *ω*2,…, *ω*m} subtract the same word in *T*1 and *T*2 to confirm the mutual anisotropy of the compound word term *t*, which is the word specified in the statement. T1 and {*q*1, *q*2,…, *qn*} are the words in *T*2. For example, in English,

*T*1: {What are your favorite sports?}

*T*2: {What kind of sports do you enjoy most?}

Combine the two statements, delete the articles and exclamations in the two statements, retain the prototype of real words, and record the same words to obtain the combined statement as follows:

*T*: {What is your favorite sport kind of you enjoy most?}

The union statement *t* is represented by a vector *s*. The word length of the joint semantic vector is the same as the number of joint sentences. At the same time, sentence *T*1 represents the joint semantic vector *S*1 and *T*2 represents the joint semantic vector *S*2 [8]. Take the words in the vector as component values. If wi is included in the semantic vector, Si is taken as 1. If it is not included in the sentence, calculate the similarity according to (1). Obtain the semantic vectors *S*1 and *S*2 corresponding to statements *T*1 and *T*2.

(3) $$\begin{array}{c} S1:\left\{1,1,1,1,1,0,8,0,8,0,0,1,0,8,0,9\right\}\text{,}\\ S2:\left\{1,0,1,0,8,1,1,1,1,1,1,1\right\}\text{.} \end{array}$$

The decimal obtained by calculation is the similarity value corresponding to the word. There are no comparative words in prepositions and auxiliary verbs such as of and do. After determining the semantic vectors *S*1 and *S*2 corresponding to statements *T*1 and *T*2, calculate the similarity between *T*1 and *T*2 according to (4) and take the alternative answer whose similarity value is greater than the set threshold as the final answer, given as follows:

(4) $$\begin{array}{c} sim\left(T1,T2\right)=\frac{S1\cdot S2}{\left\|S1\right\|\cdot \left\|S2\right\|}\text{.} \end{array}$$

**Figure 1:**
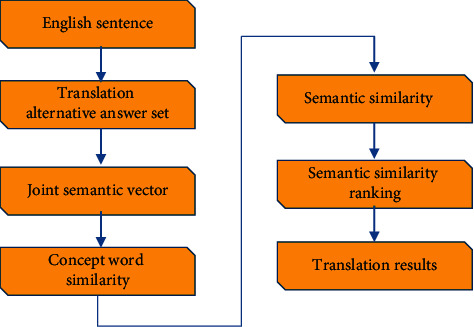
Flowchart of the English distance similarity algorithm.

The algorithm inputs an English sentence for the user, outputs all translation semantics and similarity values similar to the sentence, takes the similarity value range [0, 1], and determines the specific processing flow of the algorithm, as shown in Figure 1.

1. Put an English sentence in front of the sentence and get an answer from the translated sentence
2. Correct sentences and other answers and draw a part of speech for each word
3. Completed sentences and answers form a joint statement, which is vectorized into a set of vector lights
4. The notion of the same value is given by the semantic vector equation, and a similar value is obtained as the cosine equation
5. A similar sentence was chosen as the final answer [9, 10]

### 2.2. Semantic Analysis Intelligent Algorithm Structure

**Figure 2:**
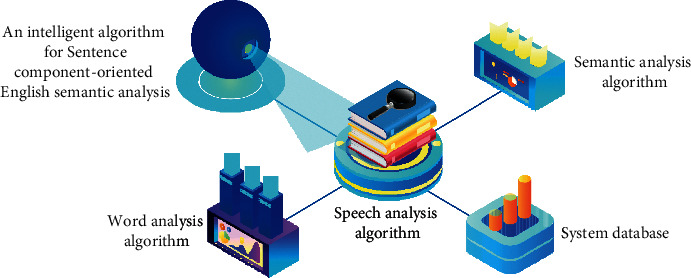
Structure of the semantic analysis intelligent algorithm.

The intelligent algorithm structure based on semantic analysis first needs to decompose the English words contained in a sentence into single words, then call the part of speech and the corresponding meaning of each word, and finally analyze the meaning of the word in the sentence in combination with the context. The structure of the semantic analysis intelligence algorithm is shown in Figure 2.

### 2.3. Word Analysis Algorithm

The main function of a word analysis algorithm is to insert text into a sentence and define words in order to provide the data for the sentence as part of the speech analysis algorithm. His work is divided into two stages.

1. Divide the statements into words, such as this is a test in English. The system divides the sentence into four words as follows: this, is, a, and test [11].
2. Determine the form of each word (word form includes the following: original form, noun plural, adjective, adverb comparative, superlative, verb third person singular, verb present form, and past form) [12]. For example, in the English sentence, I write two words last night, and the form of each word is determined as a prototype, past tense, prototype, noun plural, prototype, and prototype.

### 2.4. Sentence Part of Speech Analysis Algorithm

The main function of the sentence part of the speech analysis algorithm is to determine the speech part of the word based on the results data of the word analysis algorithm and to provide data to the line meaning analysis algorithm, and his work is divided into two stages.

**Figure 3:**
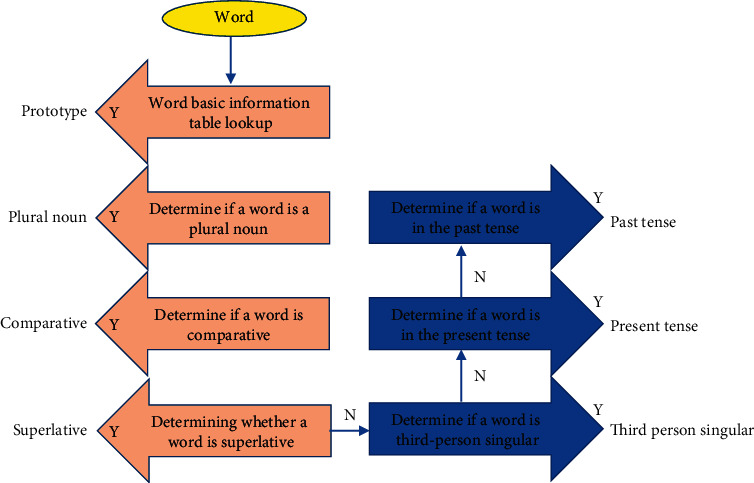
Word morphology analysis process.

1. Get all parts of speech for each word. The system obtains the part of speech of the word according to the part of speech and interpretation of the word. For example, the part of speech and interpretation of the word abuse are *n*, abuse, and bad habits [13]; *v* abuse, then all parts of speech of the word are *n* and *v*, that is, nouns and verbs. The process of word morphology analysis is shown in Figure 3.
2. Determine the part of speech of the word according to its own part of speech and the part of speech of the words before and after the word. For multipart of speech words, the algorithm scans sentences according to English grammar rules to determine the part of speech of words. For example, in the sentence the sun rises in the east, according to the grammar rules, the multipart of speech words sun and east are determined as noun parts of speech [14].

### 2.5. Sentence Semantic Analysis Algorithm

The main function of the sentence semantic analysis algorithm is to determine the main components (subject component, predicate component, the object component) and other components of the sentence according to the result information of the sentence part of the speech analysis algorithm [15]. The speech part, which is defined according to English grammar, uses a continuous piece of information to identify the first part of the sentence, then the content and nature of the sentence, and finally the part nonpredicate sentences, dots, and objects in the same way as other objects.

### 2.6. Neural Network Algorithm

**Figure 4:**
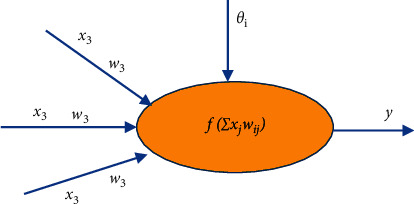
Basic structure of neurons.

An artificial neural network can use mathematical methods to simulate the biological abilities of the human brain, such as memory and information processing. The research of artificial neural network mainly involves three aspects: neuron structure, neural network topology method, and network training method [16]. The basic structure of neurons is shown in Figure 4.

In the figure, *x*=(*x*1, *x*2,…, *x*t,…, *xn*) *T* is the input signal of neurons. Neurons are connected through certain weights *WJ*, and different input signals correspond to different input weights. These weights constitute the weight matrix *w* = (*w*1*j*,*w*2*j*,…,*wtj*,…,*wrj*) of the neuron. The output formula of neurons is as follows:

(5) $$\begin{array}{c} y=f\left(x_{1}\omega_{r1}+x_{2}\omega_{r2}+\cdots +x_{n}\omega_{rn}-\theta r\right)\text{,}\\ =f\left(\overset{n}{\underset{i=0}{\sum }}\omega_{ir}x_{r}-\theta r\right)\text{,} \end{array}$$

where *f* () is the activation function, which is a nonlinear transformation. By introducing nonlinear transformation into neurons, the analogy ability of neural networks can be enhanced [17]. Commonly used energies are SGN (), tanh (), sigmoid (), and others. Many neurons can receive a neural network from head to tail (using the output of the previous stage for the input of the next stage). The most commonly used method of training neural networks is BP. However, this approach to English analysis has disadvantages such as slow learning, easy access to local consensus, and poor implementation [18].

**Figure 5:**
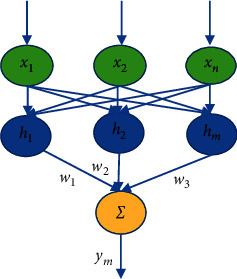
Radial basis function neural network topology.

Radial root function (RBF) neural networks are presented in this paper to overcome the negative effects of BP neural networks in English semantic analysis. The network topology is shown in Figure 5.

As you can see, the network has three layers: the login process, the layer, and the release process. The network uses a transfer topology. Compared to the functional neuron model, the RBF uses the Gaussian function to hide the latent process in the latent process.

(6) $$\begin{array}{c} hj=\exp\left(\frac{{\left\|x-cj\right\|}^{{}_{2}}}{2b_{j}^{2}}\right)\text{,}\\ c=\left[cij\right]=\left[\begin{array}{ccc} c11 & \cdots & c1m\\ \vdots & \ddots & \vdots \\ cn1 & \cdots & cnm \end{array}\right]\text{,}\\ b={\left[b1,\cdots ,bm\right]}^{T}\text{,}\\ ym=\overset{m}{\underset{j}{\sum }}\omega jhj\text{,} \end{array}$$

where *c* is the control of the Gaussian base function of the *j*-th hidden layer, and *b* is the width of the Gaussian base function. Bring the RBF network output closer to the best output for RBF network use [19].

Training in the RBF network using gradients. The special procedure is as follows: first, identify the network error. In this form, the term square deviation is used as a function to measure network error.

(7) $$\begin{array}{c} E\left(t\right)=\frac{1}{2}{\left(y\left(t\right)-ym\left(t\right)\right)}^{2}\text{.} \end{array}$$

Update the weight *w* to obtain the following function:

(8) $$\begin{array}{c} \omega j\left(t\right)=\omega j\left(t-1\right)+\Delta \omega j\left(t\right)+\delta \left(\omega j\left(t-1\right)-\omega j\left(t-2\right)\right)\text{,}\\ \Delta \omega j\left(t\right)=-\gamma \frac{\partial E}{\partial \omega j}=\gamma \left(y\left(t\right)-ym\left(t\right)\right)hj\text{.} \end{array}$$

Update the width *b* to get the following function:

(9) $$\begin{array}{c} bj\left(t\right)=bj\left(t-1\right)+\Delta bj\left(t\right)+\delta \left(bj\left(t-1\right)-bj\left(t-2\right)\right)\text{,}\\ \Delta bj\left(t\right)=-\gamma \frac{\partial E}{\partial bj}=\gamma \left(y\left(t\right)-ym\left(t\right)\right)\omega jhj\frac{{\left\|x-cj\right\|}^{2}}{b_{j}^{3}}\text{.} \end{array}$$

Update the central coordinate *c* to obtain the following function:

(10) $$\begin{array}{c} cji\left(t\right)=cji\left(t-1\right)+\Delta cji\left(t\right)+\delta \left(cji\left(t-1\right)-cji\left(t-2\right)\right)\text{,}\\ \Delta cji\left(t\right)=-\gamma \frac{\partial E}{\partial cji}=\gamma \left(y\left(t\right)-ym\left(t\right)\right)\omega ihj\frac{x-cji}{b_{j}^{2}}\text{.} \end{array}$$

**Figure 6:**
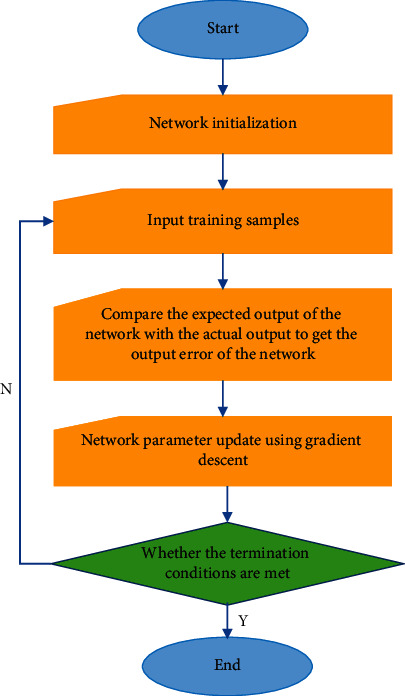
Training flow of the RBF network.

*γ* is the learning rate of the model and *δ* is the momentum factor of the model. The training process of the RBF network is shown in Figure 6.

The diagram above shows the standard flow using the RBF network. First, we need to start the required network and set up the appropriate network. Second, the model training model is included in the presentation network. To reduce the slope of the network and correct conflicts, compare the benefits of the network with the best results to achieve the best results. RBF training is a continuous process until the network output approaches the optimal output.

## 3. Design of the English Semantic Analysis System

**Figure 7:**
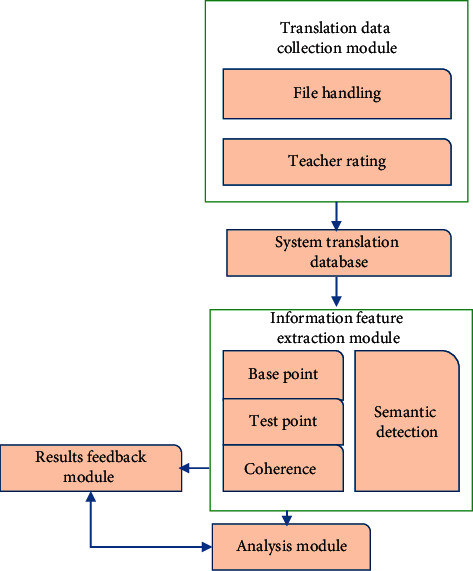
Structure of the English semantic analysis system.

The English translator automatically stores the received data in the system database and returns the results to the users by analyzing the text, deleting the data features, and checking the language and meaning in a specific context. Designing hierarchical models as required by the English language foundation includes four functions: translation, document characteristics, data structure, analysis, and feedback. The structure of the English semantic analysis is shown in Figure 7.

Take the sentence “I drew a picture yesterday.” as an example and introduce each piece of the structure separately. Basic vocabulary information is as follows: according to the syllabus, store the part of speech of the required vocabulary, pronunciation, and phonetic symbols in different environments and other information in the database. Take drew (the past tense of draw) in the example statement as an example, and its basic information is shown in Table 1.

Special vocabulary information: in English, the plural of most nouns, the past tense and past participle and present tense of verbs, and the comparative and superlative of adjectives have certain formation rules, but the above information of some words is inconsistent with common ones and has particularity. Special vocabulary information is shown in Table 2.

When the word “draw” is used as a verb, the present participle and the third person singular conform to the general rules of English vocabulary deformation, but the past tense and past participle are obviously different from the conventional ones. In order to realize the intelligent algorithm of semantic analysis more accurately, such vocabulary should be stored separately when building the database.

## 4. Method Implementation

### 4.1. Data Processing

Chapter 1 examines and discusses the RBF network design and training. It was then evaluated for its usefulness in English analysis. English consists of letters. Compared to other languages, there are only 26 English letters. The exchange rate is simple English characters that can be recognized directly by a neural network. The language used in this form is Englishhnd, which contains characters used in English and Canadian. These include Latin characters in English (excluding accents) and Arabic numerals. The log file contains 64 types of characters (0∼9, a∼*z*, and A∼Z). These include 7,705 characters from desktop images, 3,410 characters from tablet computers, and 62,992 characters from computer fonts.

**Figure 8:**
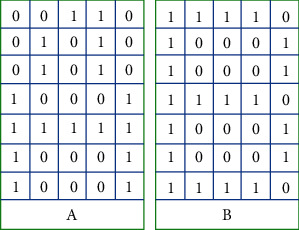
Representation of letters A and B.

Since this paper only recognizes English characters, first, the characters corresponding to “0∼9” in the dataset are screened out. Second, for each English letter, it is expressed as a 7 × 5 square by digitization. The representation of letters A and B is shown in Figure 8.

52 different representations. Next, in order from line to line, we make 35 vectors of the same length for different labels. “A” and “B” are usually expressed as follows:

*A* = [00100010100101010001111111000110001].

*B* = [11110100011000111110100011000111110].

After digitizing characters, the collected images are often disturbed by noise in the actual English character recognition. In this paper, Englishhnd is applied to the actual scene. The operation of adding noise can be realized by the randn function in MATLAB software.

### 4.2. Simulation Results

**Figure 9:**
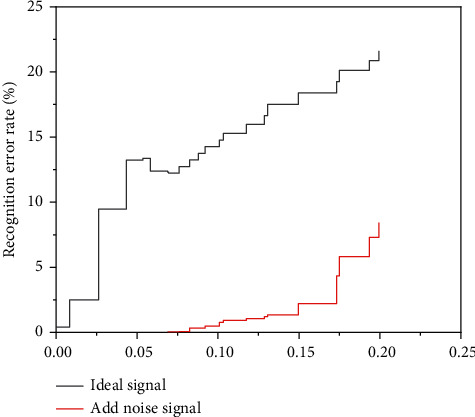
Variation of a recognition error rate of the BP network for the training set with the noise level.

**Figure 10:**
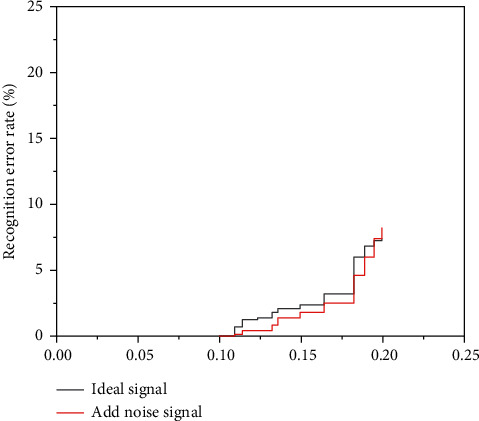
Variation of a recognition error rate of the BRF network for the training set with the noise level.

The variation of a recognition error rate of BP and BRF networks for the training set with noise level is shown in Figure 9 and Figure 10.

The diagram lines show the change in error with the measurement dataset in addition to the noise after training with the most appropriate signal. The dashed lines indicate an error change after the sound has been applied. It can be seen from the product line that network knowledge errors will be better after the addition of noise to train the BP network with the most suitable signal without noise. When the training characters are used aloud, the dashed line in the figure shows that the network is less exposed to noise during the experiment. As a result, popular graphs have a significant impact on the BP network. The network can only receive better when the meter is silent. As can be seen from the lines in the figure, when silent data are used to train the BRF network, when the average data is equal to 0.1, the error detection capability of the network changes significantly. During intensive data preparation, network performance degrades when the average test data noise exceeds 0.1. Because of the similarities between dashed lines and product lines, BRF networks are less susceptible to known operational noise and have stronger noise protection than BP neural networks.

After the noise is added to the training data, the test results of the test set are shown in Table 3 when the noise level of BP and BRF networks is 0.1.

Under the same network parameters, training data, and test data, the recognition accuracy of the BRF network for English characters can reach 96.35%, which is 7.79% higher than 88.56% of the BP network; the AUC of the BRF network reaches 0.89, which is closer to 1 compared with 0.72 of the BP network. The test results are in good agreement with the antinoise curve test results of the figure.

### 4.3. Application Realization

**Figure 11:**
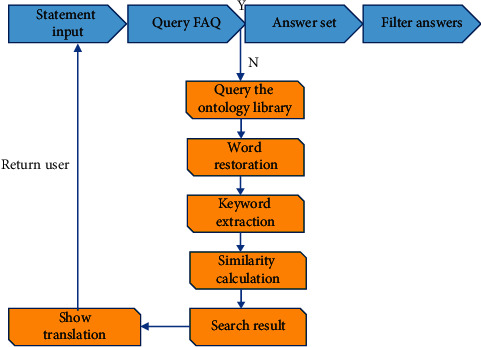
The specific process of the English semantic analysis system.

The system takes MySQL database and knowledge question bank as the data management system. The common word dictionary and WordNet semantic dictionary are used as data tables, and the hierarchical design is carried out in the Eclipse platform architecture to make the specific flowchart of the system, as shown in Figure 11.

The corpus test results are shown in Table 4.

The calculation formulas of recall rate and accuracy rate in the corpus test results are as follows:

(11) $$\begin{array}{c} R=\frac{Number\;of\;times\;to\;output\;correct\;translation}{Total\;sentence\;pairs}\times 100\%\text{,}\\ P=\frac{Number\;of\;times\;to\;output\;correct\;translation}{Output\;translation\;times}\times 100\%\text{.} \end{array}$$

Among them, the accuracy and calling rate of test Case 3 are lower than those of the other two. This is because Case 3 is mainly a news subject, intertwined with narrative text and explanatory text, there are many changes in tense and some errors. Therefore, it is necessary to increase the in-depth research on the limited field, scene and sentence pattern recognition, as well as the research on the law of sentence cohesion.

The following is an analysis of the tense problem often existing in English translation. In the transformation process of machine translation based on semantic language, the result is more the original form of English verbs. For example, the process of translating the English sentence “The will investigate this work” into Chinese based on the machine translation system is as follows:

(1) Expand the process of Chinese sentence: code the semantic unit “the will investigate this work” ⟶ 1 (the, this work) ⟶ 1 (they, 2 (work)) ⟶ 1 (2, 3 (4)).
(2) The semantic analysis process is as follows: 1 (2, 3 (4)) ⟶ “investigate (they, the (work))” ⟶ “investigate (they, the (work)) 1” ⟶ “they (2) investigate the (work) (3)” ⟶ “they (2) investigate the work (4)” ⟶ “they investigate the work.”
  1. The regular expression used in the sentence part of the speech analysis algorithm to judge the form of English words
  2. In the part of speech analysis algorithm, regular expressions are used to determine the part of speech of multipart of speech words, and part of regular expressions of multipart of speech words are determined.
  3. The regular expression used in the sentence semantic analysis algorithm determines the sentence component from the word part of the speech string, and the part of the regular expression is used to determine the sentence component.

### 4.4. Application Examples

Using the English semantic analysis algorithm based on sentence components, we have successfully realized the automatic scoring of English Chinese translation subjective questions and applied it to the “College English web examination system” of our college. The “College English web examination system” is a web-based English computer examination system independently developed by our college. In addition to automatically scoring objective questions, such as multiple-choice questions, blank-filling questions, and judgment questions, the examination system can also automatically score simple English Chinese-English subjective questions. An English semantic sentence analysis algorithm based on sentence structure involves sentence analysis, speech section and sample structure, sentence analysis of the meaning of the observer's answers, sampling, and the meaning of the respondent's answer, and then comparing the two sentences. Obtain similarities between the respondent's answer and the respondent's answer. For example, there is an example answer: I wrote a letter yesterday. Candidate's answer: I wrote a letter yesterday. The range of the two sentences and their similarities are shown in Table 5.

Taking the sentence “I drew a picture yesterday.” as an example, each module and implementation method of the system are analyzed. Lexical analysis is the process of separating each word in a sentence and analyzing its part of speech and meaning. In the sentence “I drew a picture yesterday,” it is first divided into the following words: “I,” “drew,” “a,” “picture,” “yesterday.” Retrieve all the meanings of each word in the database, such as “picture”: *n*. photos, films, descriptions, pictures; Vt. imagine, draw, take pictures. After the retrieval is completed, prepare for the next sentence part of speech analysis. Part of a speech analysis sentence is usually a word analysis. Identify the part of speech in this sentence by first finding the meaning of each part of speech and then saying before and after the word as the information is received. Sentences are the process of identifying the meaning of a word or phrase and completing a sentence to determine the meaning of a sentence, such as a sentence, beforehand, and the properties of the sentence. The system, based on the results of English grammar and sentence analysis, first determines the integrity of the finished sentence and then determines the meaning and context of the sentence according to the meaning of the sentence with the help of the following instructions of the grammar. The predicate defines the role of the object in the sentence and changes the section depending on the location of the object.

## 5. Summary and Prospect

To achieve this, the Internet age requires a focus on sophisticated and fast English to reduce communication costs. In this case, a network of radial base functions is used in the field of technology to increase the efficiency of network training. This paper focuses on the activities of the BP network and the BRF network to recognize behaviors in different voices, proving that the BRF network has a better understanding of event noise. Based on the case design, this document defines the rules and relevance of semantic extraction. Learning the algorithm of similarity of sentences in English and obtaining numbers similar to vector elements using the vector spatial design standard showed the differences between sentences and parts of speech, syntax, tense sentences, and different meanings. In terms of efficiency and functionality, the system can improve the quality of English translation quality and improve system performance.

## Figures and Tables

**Table 1:** Basic information of vocabulary (draw as an example).

Vocabulary	Phonetic symbols	Part of speech	Word meaning
Draw	(drɔ:)	vt. and vi.	Painting and painting
		vt. and vi.	Drag and pull
		vt. and vi.	Pull out
		n.(S)	Draw lots
		n.(C)	Draw and tie
		n.(C)	An attractive person or thing

**Table 2:** Special vocabulary information (draw as an example).

Vocabulary	Past tense	Past participle	Present participle	Third person singular
Draw	Drew	Drawn	Drawing	Draws

**Table 3:** Test results of the test set.

	BP network	BRF network
Recognition accuracy	88.56%	96.35%
AUC	0.72	0.89

**Table 4:** Corpus test results.

Type	Total number of sentence pairs, *N*	Recall rate, *R* (%)	Accuracy, *P* (%)	Displays the number of failures
Test case 1	400	95.25	96.36	7
Test case 2	361	91.46	91.26	3
Test case 3	186	70.35	72.64	5

**Table 5:** Various components of the two sentences and their similarity.

Type	Subject component	Predicate element	Object component	Other ingredients
Standard answer	I	Wrote	A letter	Last night
Examinee answer	I	Write	Letter	Last night
Similarity of components	100%	50%	50%	100%
Sentence similarity	75%			

## Data Availability

The data used to support the findings of this study are available from the corresponding author upon request.

## References

[1] Lu Q. (2021). A new project-based learning concept in English writing. *International Journal of Emerging Technologies in Learning (iJET)*.

[2] Zeng G. (2022). Intelligent Test Algorithm for English Writing Using English Semantic and Neural Networks. *Mobile Information Systems*.

[3] Winter T., Le Foll E. (2022). Testing the pedagogical norm. *International Journal of Learner Corpus Research*.

[4] Li Y., Xiao G. (2019). Chinese college students’ English writing anxiety and its related factors: a survey study. *International Journal of Social Science and Education Research*.

[5] Luo Y. (2022). Research on multifeature intelligent correction of spoken English. *Computational Intelligence and Neuroscience*.

[6] Yang L., Liu W. (2021). Design of English intelligent simulated paper marking system. *Complexity*.

[7] Shi H., Shi C. (2022). Intelligent interactive English teaching system for engineering education. *Advances in Multimedia*.

[8] Zhang B. (2022). Unsupervised English Intelligent Machine Translation in Wireless Network Environment. *Security and Communication Networks*.

[9] Li X. (2022). Intelligent Interactive English Teaching Discrete Data Modeling and Simulation. *Scientific Programming*.

[10] Huo Y. (2019). Analysis of intelligent evaluation algorithm based on English diagnostic system. *Cluster Computing*.

[11] Wang X. Y. (2022). Intelligent English translation and optimization based on big data model. *Journal of Sensors*.

[12] Miao Y., Liu H., Gu S. (2022). English speech feature recognition-based fuzzy algorithm and artificial intelligent. *Wireless Communications and Mobile Computing*.

[13] Hilal A. M., Al-Wesabi F. N., Abdelmaboud A., Hamza M. A., Mahzari M., Hassan A. Q. A. (2022). A hybrid intelligent text watermarking and natural language processing approach for transferring and receiving an authentic English text via Internet. *The Computer Journal*.

[14] Xu Z., Kamruzzaman M. M., Shi J. (15 April 2022). Method of generating face image based on text description of generating adversarial network. *Journal of Electronic Imaging*.

[15] Ghareeb S., Hussain A. J., Al-Jumeily D., Al-Jumeily K, Baker Al S, Khalaf M. (2022). Evaluating student levelling based on machine learning model’s performance. *Discover Internet of Things*.

[16] Fitria T. N. (2021). Grammarly as AI-powered English writing assistant: students’ alternative for writing English. *Metathesis: Journal of English Language, Literature, and Teaching*.

[17] Nurmalia L., Nuraeni C. (2021). An analysis of errors in English writing: a case study the third semester students of ubsi. *JURNAL ARBITRER*.

[18] Mohammadi T., Mustafa H. R. (2020). Errors in English writing of esl/efl students: a systematic review. *Theory and Practice in Language Studies*.

[19] Yumin W., Lijuan J., Jingxuan Z. (2020). An evaluation of the assessment measure for novice l2 learners’ English writing. *Linguistics and Literature Studies*.

